# A neural network based approach to classify VLF signals as rock rupture precursors

**DOI:** 10.1038/s41598-022-17803-x

**Published:** 2022-08-12

**Authors:** Adriano Nardi, Alessandro Pignatelli, Elena Spagnuolo

**Affiliations:** Istituto Nazionale di Geofisica e Vulcanologia, Via di Vigna Murata 605, 00143 Rome, Italy

**Keywords:** Geophysics, Seismology

## Abstract

The advent of novel technologies revealed that other geophysical signals than those directly related to fault motion could be used to probe the state of deformation of the Earth's crust. Electromagnetic signals belonging to this category have been increasingly investigated in the last decade in association to natural earthquakes and laboratory rock fractures. These studies are hampered by the lack of continuous recordings and a systematic mathematical processing of large data sets. Indeed, electromagnetic signals exhibit characteristic patterns on a specific frequency band (the very low frequency, VLF) that correlate uniquely with the paroxistic rupture of rocks specimens under uniaxial laboratory tests and were also detected in the atmosphere, in association to moderate magnitude earthquakes. The similarity of laboratory and atmospheric VLF offers an unique opportunity to study the relation between VLF and rock deformation on at least two different scales and to enlarge the dataset by combining laboratory and atmospheric data. In this paper we show that the enlarged VLF dataset can be successfully used, with a neural network approach based on LSTM neural networks to investigate the potential of the VLF spectrum in classifying rock rupture precursors both in nature and in the laboratory. The proposed approach lays foundation to the automatic detection of interesting VLF patterns for monitoring deformations in the seismically active Earth’s crust.

## Introduction

Seismic sequences are characterized by a seemingly irregular occurrence of earthquakes along main faults. This irregularity challenges our understanding of the physical processes governing the seismic cycle and represents a serious limitation in the modeling strategies for predictive purposes. More information can be achieved by observing continuously the rock volume which contains seismogenic faults as rutinariely done by monitoring deformation at surface or ground (and ocean) motion. Along with these signals other geophysical observables may contain relevant constraints on transient phenomena coheval to the deformation such as geochemical signatures of permeability and temperature changes (e.g. radon or CO_2_) and electromagnetic anomalies in the ionosphere. The very low frequency (VLF) range of the electromagnetic (EM) spectrum represents a very strict and promising example of these types of geophysical signals that could extend our knowledge about transient phenomena associated with the deformation of the Earth’s crust and the seismic cycle. Anomalies in the propagation of the signals of VLF radio stations have already been studied in the past as a precursor to the earthquake occurrence^1–4^. Although the source of these signals and their propagation through the atmosphere is not conclusive, the use of VLF signals as possible earthquake precursors has been widely investigated also in the laboratory with experiments on rock samples (e.g.^5–7^). Possible mechanisms of generation and propagation are summarized and discussed in previous literature (e.g.^8,9^). Limitations to the study of the natural VLF signals have been: 1. the non-systematic data acquisition due to a limited data storage and/or data transmission through the network; 2. processing of large data set, due to the high acquisition rates which are of the order of tens of kHz; 3. the fact that the study of the electromagnetic signal was mainly focused on the observations of narrow EM bands. These three limitations hampered the research of EM signals as possible detectors of transient geophysical signals. However, EM signals are actually sensitive to transient phenomena: the sensitivity of the LF signals becomes apparent for seismic events with magnitude M ≥ 5.5^10^, whereas in the case of VLF signal this threshold lowers to M ≥ 4.5^6^. Nardi et al.^6^ suggested that three VLF signals were correlated with a magnitude M ≥ 4.5 earthquake, within an average of 3.6 days after the appearance of characteristic signals (Orderly Impulsive Signals, hereafter OIS) in the VLF spectrum and within 270 km maximum distance from the survey site, during an observation period where no other relevant earthquakes have occurred.

In this paper, we will use both laboratory signals and atmospheric signals to show that OIS can be used to effectively train a neural network and classify VLF signals as possible precursors of rock ruptures in the laboratory. Given the similarity of OIS signals in the laboratory and in the atmosphere we suggest that the same network, trained on both natural and laboratory signals can be used to detect transient deformation phenomena in the Earth’s crust.

### VLF spectral patterns detected in the laboratory and in the atmosphere

Experimental observations on EM-VLF signals have been conducted during rock deformation under uniaxial compression with the same equipment and the same sampling frequency of 44.1 kHz also used in on air monitoring. A 40 cm long, wide band active antenna has been designed and used to record the electric component of the EM radiation in the extremely low frequency (ELF) and very low frequency (VLF) ranges, precisely in the band 0.8–12 kHz. In the laboratory, the antenna was positioned horizontally at a distance of 10 cm from the sample. The direct transformation of the EM signal into electrical signal in the acoustic frequency band also offers the advantage to allow memorizing and analyzing the data using a common sound card, the ‘‘wav’’ file format and existing software in the field of acoustics. Still in the laboratory, to record the reference acoustic signal of the cracks, we used an omnidirectional preamplified microphone Accord PA196, with a sensitive band 0.05–16 kHz^7^.

**Figure 1:**
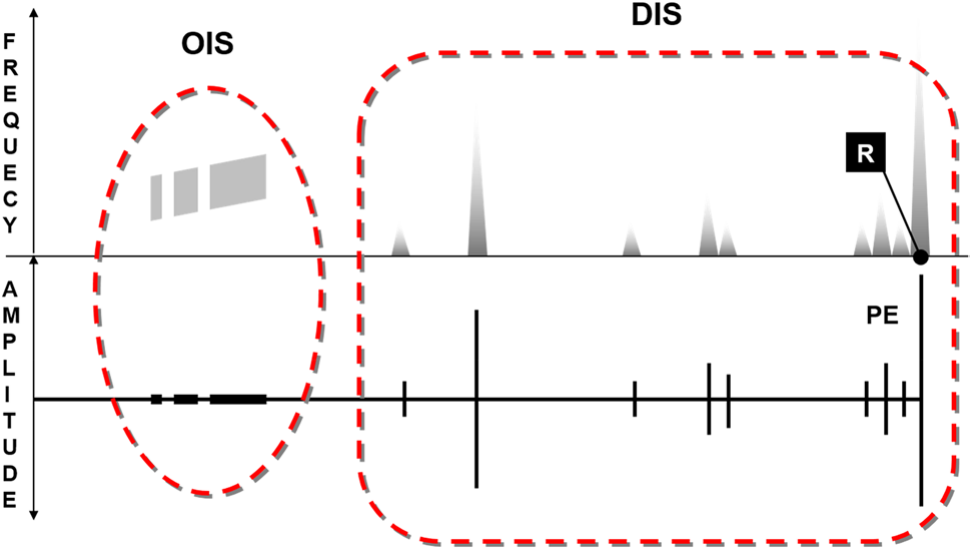
OIS and DIS signals observed in the laboratory. Spectrogram (top panel) and oscillogram (bottom panel) compared. Schematic diagram of characteristic patterns. *OIS* Orderly Impulsive Sequences, *DIS* Disorderly Impulsive Sequences, *PE* paroxysmal emission and R rupture of the rock sample. On the spectrogram they compose a uniform band centered over the average pulses frequency. (modified from^7^).

**Figure 2:**
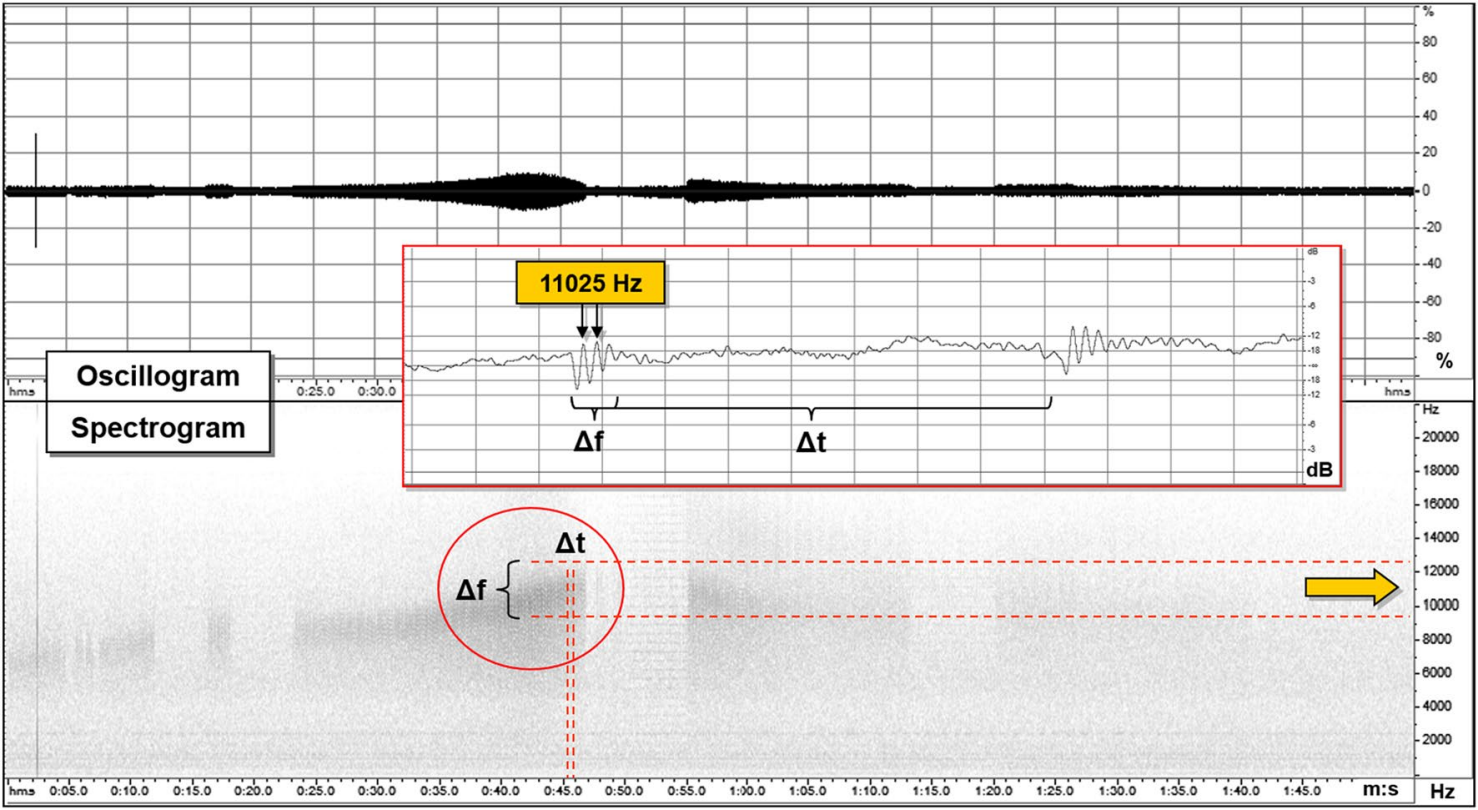
Example of LABset OIS signals. Oscillogram (top panel) and spectrogram (bottom panel) show a LAB signal consisting of regular repetitions of similar pulses. The insert is an enlargement of the oscillogram in a time window of 6 ms showing the periodic oscillation (with a frequency of ∼11 kHz) and the repetition of the signal every ∼3.5 ms (modified from^7^).

**Figure 3:**
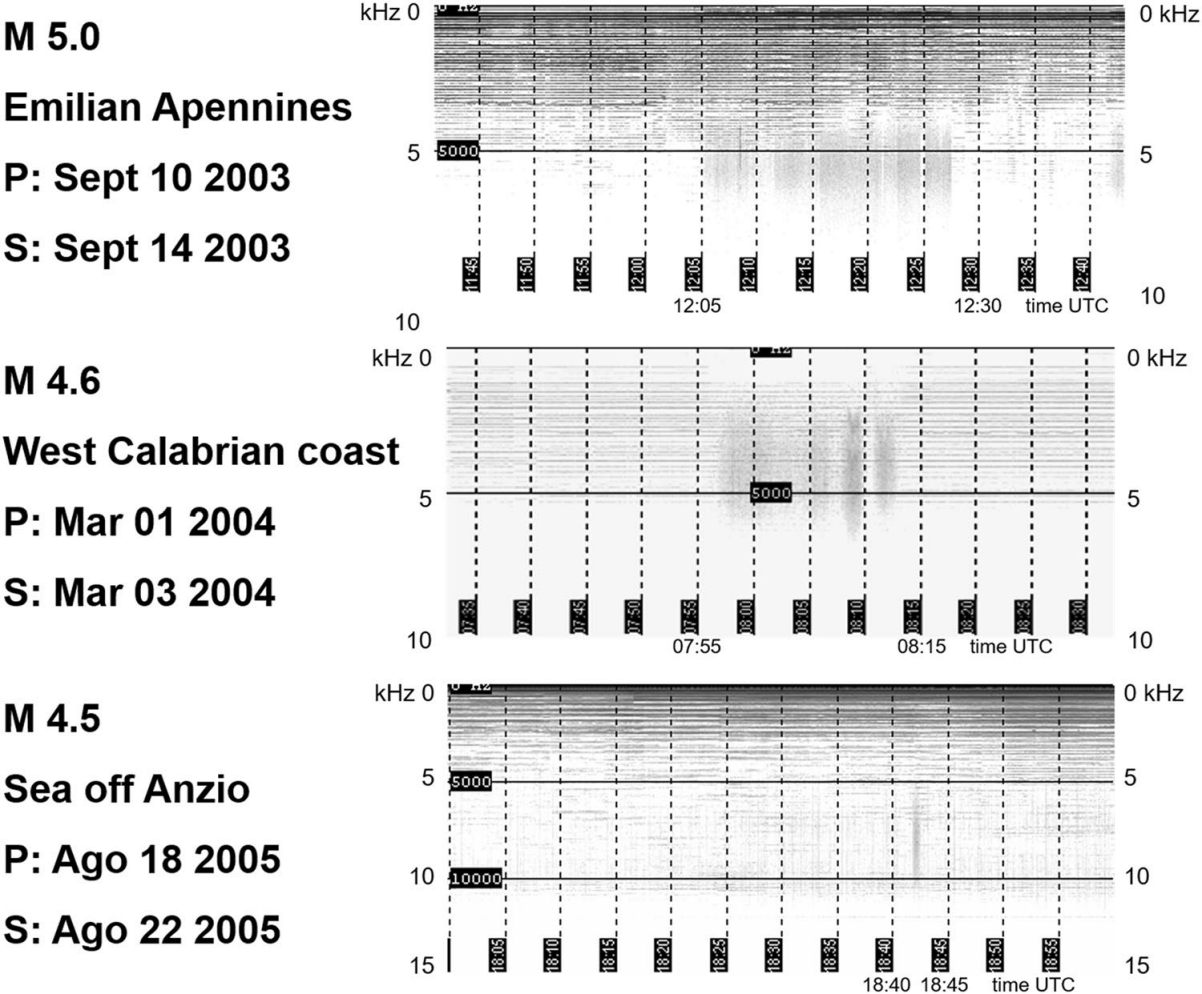
Example of AIRset OIS signals. Spectrograms of AIR signals show a regular repetition of pulses here documented in association to the three moderate magnitude seismic events labeled in figure. *P* month, day and year of the VLF recording and *S* month, day and year of the origin time of the associated seismic event. Modified from^6^).

**Figure 4:**
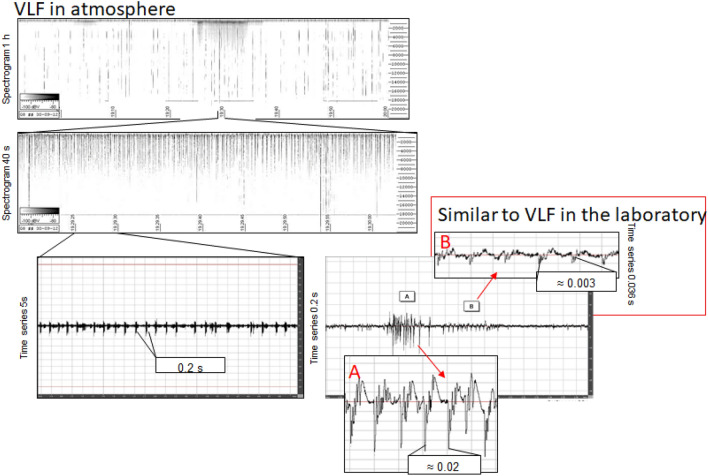
Spectrograms and temporal series recorded in the atmosphere at Serramazzoni (MO) on Sept 30th 2012 19:30 UTC and possibly associated to the M 4.5 event occured on Oct 3rd 2012 4:41 UTC at 100 km from the recording station. The 1 h spectrogram shows anomalous signals and the enlargement in inserts shows A and B type “interesting” patterns.

**Figure 5:**
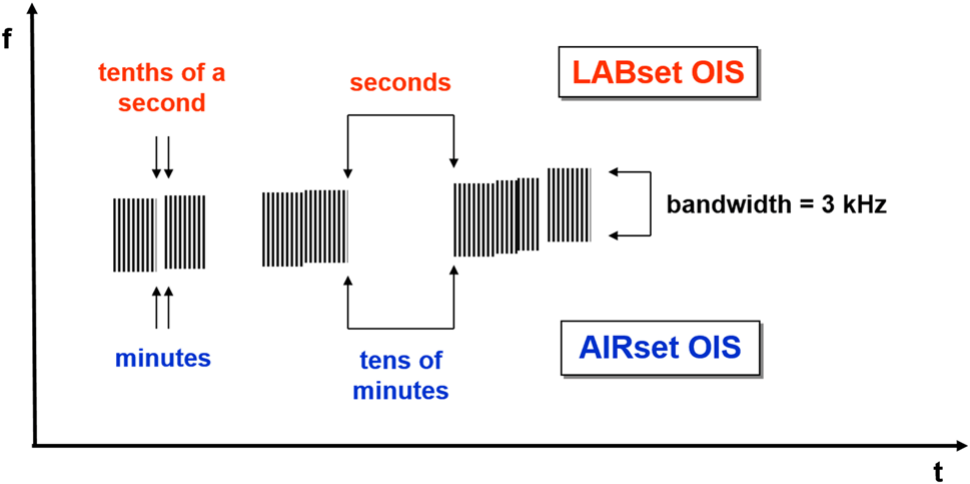
Difference between the LABset OIS and the AIRset OIS. The black columns represent the trains of pulses seen in a hypothetical spectrogram. The duration of trains and the temporal separation between trains tend to remain almost equal between LABset and AIRset.

The first recognition of EM signals as a precursor in the atmosphere was performed by Warwick et al.^5^ who also conducted experiments on rocks to verify the correlation of EM emission with rock deformation. Experimental evidence^7^ documented that VLF signals associated with rock fracturing occur in the form of mainly two impulsive event types occurring before, during, and immediately after the rupture episode. The observed signals, which are visible both in the amplitude and frequency domain, have been divided into two groups (Fig. 1): a first one made of the so called Orderly Impulsive Sequences (OIS) and the second one, the Disorderly Impulsive Sequences (DIS), which are irregular impulsive signals appearing after the OIS and exhibiting a proximal emission (PE in Fig. 1) close to the rupture (R) episode. In this work we will focus on OIS emissions because they are characterized by high frequency almost identical micro-impulses appearing at regular time intervals and composing “pulse trains”. On the spectrogram of a laboratory signal (Fig. 2) they compose a uniform band centered over the average pulses frequency (11,025 Hz, indicated by a yellow arrow on the spectrogram). In this case the signal appears in the spectrogram with an ascending frequency suggesting that the average frequency is also increasing. The signal intensity and the number of OIS pulses are influenced by mechanical and structural characteristics of the material, by the presence of fluids, and to a lesser extent, by the lithology. OIS signals are the most easily sampled and recognized in continuous monitoring work. Importantly, natural observation of VLF by the italian monitoring network “Cassandra”, documented at least three events (Fig. 3) characterized by signals fitting OIS laboratory models^6^. The three spectrograms show one frequency band centered on an average frequency with bandwidth Δf. The spectral pattern is similar to LABset OIS (cfr. Fig. 2 and 3, see also Fig. 4). Spectrograms in Fig. 3 show the regular repetition of similar pulse trains with a frequency band centered on the average frequency of the pulses as found in laboratory experiments (cfr. Fig. 2 and 3). In summary, OIS signals manifest similar patterns at different scales as shown in Fig. 4 for a spectral pattern recorded in Serramazzoni and associated with a seismic event of magnitude M = 4.5 that occurred on October 3rd, 2012 at 4:41 UTC (Universal Time Coordinated). Inserts in Fig. 4 evidence the typical patterns (A and B) recognized by enlarging the spectrogram of natural VLF emission and zooming in with a temporal window of 0.2 s. Signal type B is similar to the recording of VLF in the laboratory and consists of trains which repeat with a period of around 4 ms. A and B type signals serve to instruct the neural network algorithm for pattern recognition of VLF atmospheric signals. Compared to OIS signals of Fig. 2, the main difference between OIS from the laboratory (LABset) and those recorded in the atmosphere (AIRset) is the time scale, which lasts for few minutes for AIRset versus a few seconds in the case of LABset as schematized in Fig. 5. The bandwidth in both cases is about 3 kHz and corresponds to the variability of the single pulse period. The black columns represent the trains of pulses seen in a hypothetical spectrogram. The duration of trains and the temporal separation between trains tend to remain almost equal between LABset and AIRset (respectively about 1.2 ms and about 4 ms). This similarity can be used to better identify VLF signals and characterize its phenomenology under controlled laboratory conditions, on a temporal scale which allows us to acquire a statistically consistent database of events, and to test the correlation between the VLF signals detected in the atmosphere with the deformation of rock volumes. VLF spectral patterns appear to be correlated to acoustic emissions caused by micro and macro fracturing processes preceding and leading to sample failure. Moreover, the magnitude threshold of VLF acts as a natural filter on moderate to large seismic events suggesting that VLF spectral patterns have the potential to detect deformation of large rock volumes within the Earth’s crust.

### The need for a novel approach to measures and analysis: machine learning

VLF signals have been studied discontinuously and non-systematically using different measuring systems and investigating different frequency bands. Indeed, previous studies were focusing on specific frequencies whereas the evolution of the EM signals during the rupture sequence is only visible using the entire spectra of the ELF-VLF band (Fig. 1, from^7^). However, the recognition of interesting patterns in the atmosphere and thus their associations with transient natural events is largely hindered by the lack of a systematic approach to the measuring systems, the lack of continuous data over a statistically consistent temporal and spatial interval and in general, by the approach to data management. Moreover, although these patterns appear clear in the spectrogram from visual inspection, designing a specific mathematical algorithm to find them automatically is not trivial. For the aforementioned reasons in this study we have overcome these limitations using:

1. A VLF EM monitoring network termed as “Cassandra” which is a systematic instrumented network for VLF detection in the atmosphere. The type of antenna and the network design deployed on the national territory is described in Nardi and Caputo^7^.
2. A machine learning algorithm for automatic signal detection by scanning VLF spectral patterns over the huge amount of signals typically acquired at 44.1 kHz in a frequency band 20 Hz—22 kHz.

The use of machine learning (ML) techniques have been tremendously increased in the last few years with the increasingly large amount of data available. More specifically, the neural networks are used for a wide range of different purposes such as image and text recognition, speech interpretation and many other uses. The main benefits in using ML are:

1. Versatility, because by only changing input data they can solve many problems.
2. Automation, in fact once the algorithm is designed, the only requirement is to provide enough data to let the machine learn how to solve the problem and then the system will be able to autonomously and automatically solve the same problem in similar different situations.
3. Objectivity, as machines are not “biased by emotions”. For example, medical research procedures require double blinded protocols to avoid biases. When using machine learning techniques there are no specific protocols to be fit.

A particular case of machine learning techniques is represented by neural networks (NN). The neural network “architecture” is inspired by the structure of the primate brain cerebral cortex, in order to learn increasingly abstract features of the input and best support the desired output^11,12^. The main advantage in using neural networks as compared to other types of machine learning techniques is that features relevant for the prediction do not need to be selected in advance as they are automatically found by network training. In this work, we designed the NN to detect the most typical signal patterns of the natural atmospheric noise as well as OIS patterns in the VLF band. NN was trained on VLF signals which preceded rock failure in the laboratory under uniaxial tests as well as on natural VLF signals which occurred a few days (< 5 days) before a moderate magnitude earthquake (M > 4.5) exploiting the apparent scale invariance of the VLF OIS. Laboratory OIS can be used effectively to enlarge the data set and to train the neural network for the detection of OIS signals in the atmosphere.

### Neural network architecture and application

**Figure 6:**
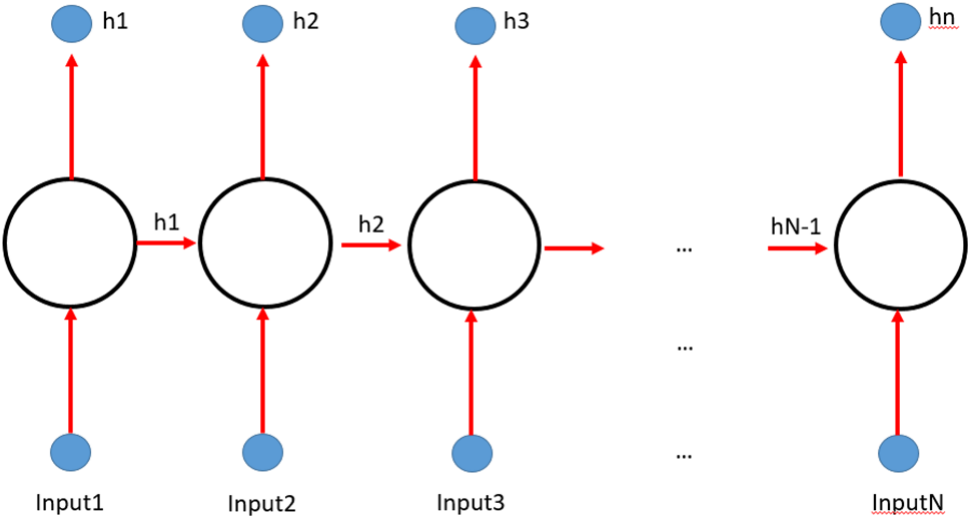
RNN main architecture. In a recurrent neural network structure (RRN) each neuron is influenced by the input data and previous ones.

We have implemented an Artificial Neural Network (ANN) architecture whose structure is designed to deal with time series. More specifically, such architectures are based on recurrent neural networks (RNN, as suggested by Pascanu et al.^13)^. The basic idea of RNN shown in Fig. 6 is that a neuron output is not influenced just by the convolution between its input and its activation function but also by the output of its adjacent neuron. A RNN network has a “short term” memory as “previous neurons’ memory rapidly vanishes between neurons. To overcome this limitation, different base structures called Long Short-Term memory (LSTM) blocks have been used in literature^14–16^. Basically, LSTM blocks are an update of RNN ones to keep more sequence memory. An example of a LSTM is explained in Yildirim^17^. The LSTM blocks replace the neurons in Fig. 6 to set up the same structure using a different block as shown in Yildirim^17^. The main limitation of LSTM networks is that only “past” input in the sequence can affect the “future” neurons. That’s why a new version of LSTM called “bidirectional LSTM” (BI-LSTM) has been designed to put together a forward and a backward LSTM. Both networks are connected to inputs and to outputs. A general classification shallow LSTM neural network consists of an input layer with a number of neurons as the input sequence, a LSTM blocks layer, a fully connected layer (with a number of neurons as the output classes) a softmax layer and a classification layer. As described by Sagheer and Kotb^18^, it is possible to create a deep LSTM neural network architecture, adding more hidden LSTM layers after the first one.

In this case study, neural network application is very simple: after careful data collection, we have signal sequences, some of them classified as rock rupture precursors (“RUPTURE”) and some of them not (“QUIET”). Signals composing the background noise, including the anthropic signals and natural signals such as sferics, are all belonging to the category QUIET.

It is worth noting that the LABset OIS data labeled as rock rupture precursors showed spectral patterns similar to the AIRset OIS events collected a few days before the Italian earthquakes listed in Fig. 3.

A deep BI-LSTM neural network with 1000 hidden units has been trained in order to fit the known classification so implicitly understanding the most important features and cut offs to split the “potential events” to “not events”.

## Materials and methods

### LABset data

Our case study dataset is composed of signals acquired using both laboratory experiments and an experimental monitoring network. The first dataset, hereafter “LABset”, are VLF signals generated by 36 rock samples ruptured under uniaxial compression. The 36 samples belong to 14 different lithotypes including porphyry, granites, basalts, peridotites, limestones and concrete. To detect VLF signals during laboratory tests we have used the ELF-VLF spectrum sampled at the frequency of 44.1 kHz. Detecting and discriminating DIS and OIS from the background noise, consisting of natural (e.g. atmospheric) and artificial (e.g. electromechanical laboratory noise) signals was a complex procedure requiring a different type of spectrum analysis for OIS and DIS. Therefore, in this first monitoring phase, it was chosen to only focus on the OIS type for reasons explained below. OIS emissions are regular (see also description in “VLF spectral patterns detected in the laboratory and in the atmosphere”), the pulse variability in the period determines a band-width of about 3 kHz (Figs.1 and 2). The regular repetition in the succession of the trains determines the uniformity of the emission that is sharply interrupted at the point where train repetition stops. Considering the time span from the beginning of the test until the rupture of the sample under a constant increment of force, the OIS manifests itself in the first half of this period.

### AIRset data

The second data set, hereafter "AIRset", are VLF signals observed in the atmosphere by the stations of the monitoring network at its earlier testing stages^6,7^. These very rare signals have been selected to have characteristics similar to those observed in OIS in the laboratory. VLF signals were observed in a frequency band ranging between 20 Hz and 20 kHz (ELF-VLF radio bands) and sampled at 44,100 Hz from a number of stations deployed in Italy along the central Apennines and belonging to the Cassandra monitoring network. The OIS signal appeared to occur a few days before (seemingly up to 5 days) a moderate magnitude seismic event (M 4.5). A very important point is that OIS are not common signals of the VLF spectrum, they do not belong to any of those signals observed even rarely in the background and were only recorded when followed by a short term (days) significant earthquake (Fig. 3). Despite these relevant observations the association between OIS and natural earthquakes is still hypothetical and needs further investigation and data for providing their association.

### The neural network approach and definitions

A machine learning method tries to predict a variable (answer or label) when some others (predictors) are known. In order to make the algorithm “learn”, the first step is to collect data including both the predictors and the answer (labeled data). Such data will feed the algorithm that will “learn by itself” if predictors show patterns (said features) able to predict the answer. This machine learning stage is named training. After the training, if successful, the algorithm is able to automatically predict the answer for any other datasets including all the provided predictors. Such a “trained algorithm” able to predict is named “model”. An example of a machine learning task may be recognizing a person's gender by using his/her bibliometric and anagraphic data. In this case, height, weight, age and other data are the predictors and “male” and “female” are the labels. In order to realize a machine learning model working we need to provide the system with already labeled data. In order to check how the model is able to “generalize” its validity for data not included into the training and avoid overfitting problem^19^, a common practice is, before doing the training, randomly choose a part of the available dataset and remove it from the training process. So, when the training has been performed, the excluded data (named test data and containing the labels we are looking for) provide a powerful source to verify the model accuracy. In fact, such data include the true labels but the user can use them as “new data” to predict the label by the trained model. The comparison between the real and the predicted labels allows the accuracy percentage computation Accuracy = matched results/total test data. Moreover, when dealing with neural networks training, a common practice is to divide data into three groups: 1. training data; 2. test data and 3. validation data. Training and test data are used as described before, validation data are used during the training process to check (in real time during the training) that there is no overfitting. In fact, such data, as test data, is not used for the learning process but sometimes, during the training, the predicted classification of such data are compared to the real output. If the training accuracy is too much higher than the validation one, then an overfitting problem is detected.

We are here using a Neural Network Architecture (ANN) consisting of a set of layers of neurons (Fig. 6). Input data is inserted into the first layer (each input data into a single neuron) and each neuron changes the input value according to a transfer function depending on the ANN used. Once each data input has been processed by the neuron it is “passed” to the next neuron’s layer where the input is a linear combination of the previous layer outputs. Such linear combinations are determined by a set of coefficients (named neuron weights). The process is iterated along all the layers to the last layer (named “output layer”) and the outputs are compared to the provided labels. According to the discrepancy between the true labels and the ones obtained a backward propagation process starts modifying the weights. Such propagation, which involves the iterative adjustment of a single parameter vector, has the goal of minimizing the differences between the observed and predicted values by Cao and Parry^20^.

A big drawback of the backward propagation process is that input data are considered independent and weights networks updates calculation is not affected at all by input sequences. This makes backward propagation of neural networks not particularly suitable for time series analysis, speech recognition or any other application where input sequence sorting does matter. That’s why some network structures dealing with time and using previous states memory have been introduced^21^ which include recurrent neural networks (RNN) (e.g.^13^) and Long short term memory (LSTM) networks (e.g.^17^).

### The confusion matrix

When dealing with machine learning, the best way to show methods accuracy is using the “confusion matrix”^19^. A confusion matrix is a table counting where the predicted classes agree or disagree with the true values. Conventionally the rows represent the true classes and the columns represent the predicted ones. The number in the intersection between rows and columns represent how many records characterized by the row true classes have been classified with the predicted one specified by the column. So the numbers along the diagonal show all the records correctly classified and the other numbers the wrong ones. In addition to general accuracy, confusion matrices indicate the accuracy of the method for each single class.

## Results

**Figure 7:**
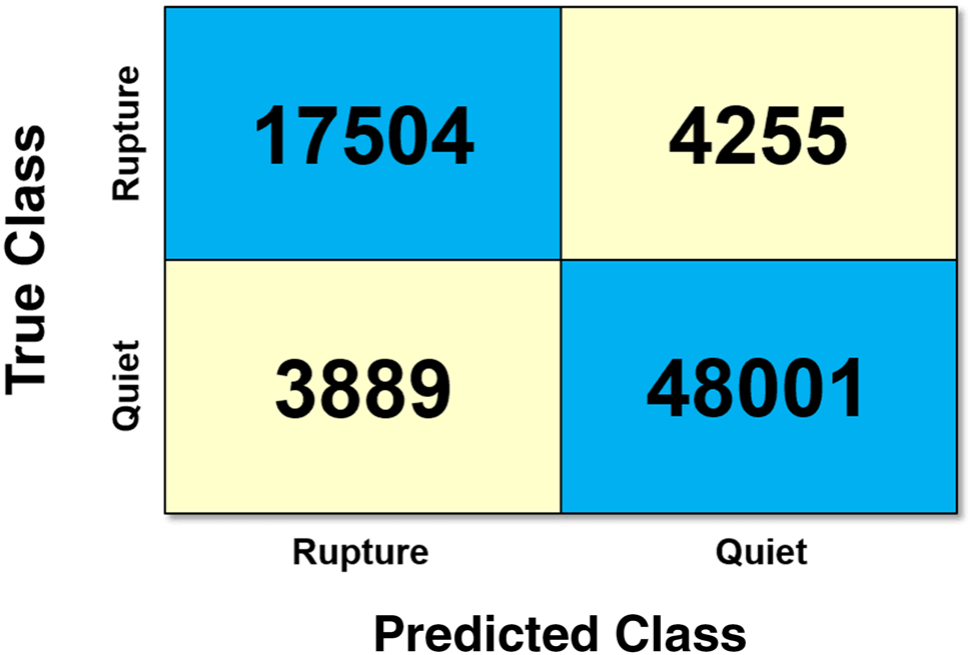
89% of the records were correctly classified. The confusion matrix reports values representing how many records characterized by the row true classes have been classified with the predicted one specified by the column.

After selecting randomly 281,949 blocks (i.e. portions of the signal), they have been used to feed and check the network and use their label to match at best the results. More specifically the training and the check has been performed as described in “The neural network approach and definitions” and 73,649 (30% of the total data) has been used for the test; 146,072 data (85% of the remaining data) have been used for the training and 25,777 data (15% of the remaining) have been used for validation. Each block is an ensemble of 1323 recorded samples. The number 1323 has been evaluated as the correct one to be sure that the signal contains the anomalous pattern. During the process we have checked that validation accuracy was similar to the training one (in other words that overfitting did not occur). Once the training has been completed, the trained network has been used to predict the classification of the 73,649 test sequences initially excluded from training (to avoid overfitting as stated in “The neural network approach and definitions”). The capability of the NN to distinguish signals of relevance from the background which are possibly related to transient geomechanical properties is very satisfactory as revealed by the confusion matrix in Fig. 7 (see also “The confusion matrix”). The accuracy from the confusion matrix (calculated according to the equation in the “Material and methods” section) shows that 89% of the records were correctly classified. This result implies that an automatic recognition of such signals by means of artificial intelligence is possible and very advantageous. The result of the confusion matrix seems to suggest that laboratory OIS can be used with success to detect atmospheric OIS signals with the advantage that laboratory signals can be acquired and integrated easily under controlled conditions.

## Discussion

The VLF signals have been proved in the laboratory to be a reliable rock rupture precursor. Indeed, recurrent and regular pulse trains defined as OIS from previous literature seem to systematically anticipate the paroxistic rupture event under uniaxial compressive tests. At the same time, OIS with similar characteristics (period and band width) have been detected in the atmosphere. The similarity of VLF-OIS in the atmosphere and in the laboratory offers the opportunity to investigate the potential of VLF signals in the study of transient phenomena on scalable temporal scales. However, the recognition of characteristic patterns in the atmosphere and thus their association to transient natural events is largely hindered by 1. the lack of a systematic approach to the measuring systems, 2. the lack of continuous data over a statistically consistent temporal and spatial interval and 3. because, even if these patterns appear clear in the spectrogram from visual inspection, designing a specific mathematical algorithm to find them automatically is not trivial. That’s the context where neural networks found applications as they are designed to detect features that can be used to classify signals as “human” would do. In this paper, we challenged such limitations to show that artificial intelligence and, more specifically LSTM neural networks, are effective “automatic detectors” for OIS. Such characteristic spectral patterns have been correctly classified by our neural network within the defined categories: precursors to rock “rupture” and “quite”. The classification has been proven effective not only for laboratory data but also for atmospheric signals recorded in association with transient natural events involving fracturing of rock volumes (e.g. earthquakes).

A couple of methodology limits have to be mentioned, mostly correlated to events magnitude and distance between events and recording stations. In fact, at the state of the art, VLF signals are not able to detect earthquakes having magnitude below 4.5 and the maximum distance range where events are detectable is not yet defined.

Despite these limitations, such results look very promising. Indeed, a precursory event can be studied and recognized when the dataset of events is statistically consistent. VLF signals cannot be studied over a statistically consistent data set because the actual dataset is typically large and relevant signals cannot be easily extracted. The proposed approach has the potential to provide a systematic study of OIS patterns because we demonstrated that the use of NN allows the automatic detection of characteristic signals in the air. The automatic detection would allow real-time monitoring, detection and extraction of characteristic patterns which would remain otherwise hidden. Moreover, we demonstrated that these OIS signals can be studied on at least two different temporal and spatial scales, at the controlled conditions of the laboratory and on natural recordings in the atmosphere, providing the opportunity to study their potential to probe the state of deformation of rock samples as well as rock volumes of the seismically active Earth’s crust.

## Data Availability

All data used to train and test neural network can be found as gitlab public project at the following link: https://gitlab.com/alessandro.pignatelli/cassandradata. Due to the big memory needed _(more than 3 Gb) they have been saved as matlab binary file and basically the file contains two matrices: “input_data” including each time series window (1323 samples) as columns and “output_data” containing the labels in oneshot format so a two rows matrix having a column for each series and the value 1 on the first row and zero on the second row if the series is a rock rupture precursor and 0 at the first row and 1 at the second row if vice-versa.

## Code availability

There is no specific code to be shared.
